# Rituximab-Associated Posterior Reversible Encephalopathy Syndrome in Severe Hydralazine-Induced Antineutrophil Cytoplasmic Antibody Vasculitis: An Unexpected Complication

**DOI:** 10.7759/cureus.103417

**Published:** 2026-02-11

**Authors:** Faiza Javed, Elizabeth Barbeau, Ozair Ziauddin

**Affiliations:** 1 Rheumatology, Franciscan Health, Chicago, USA; 2 Internal Medicine, Midwestern University Chicago College of Osteopathic Medicine, Downers Grove, USA

**Keywords:** drug-induced vasculitis, immunosupression, posterior reversible encephalopathy syndrome (pres), rituximab therapy, vasculitis

## Abstract

Patients with vasculitis are at an inherently increased risk for developing posterior reversible encephalopathy syndrome (PRES) due to disease-related factors such as uncontrolled hypertension, renal dysfunction, endothelial injury, and the need for potent immunosuppressive therapies. Although PRES remains uncommon in vasculitis cohorts, it has been reported in association with several therapeutic agents, including rituximab, high-dose glucocorticoids, and cyclophosphamide.

We present a case of hydralazine-induced dual-positive ANCA-associated vasculitis complicated by respiratory failure requiring mechanical ventilation. The patient subsequently developed PRES within hours of receiving her second dose of rituximab. PRES is a rare but serious complication associated with rituximab, particularly in patients with active or drug-induced ANCA-associated vasculitis. Vigilant monitoring is recommended in those with severe inflammation or unstable blood pressure. Any new focal neurologic deficits, seizures, visual changes, or altered mental status after rituximab infusion should raise concern for PRES and warrant immediate cessation of therapy and urgent diagnostic evaluation, highlighting the need for heightened vigilance for neurologic complications during immunosuppressive treatment of severe vasculitis.

## Introduction

Posterior reversible encephalopathy syndrome (PRES) is a clinical syndrome characterized by acute neurologic symptoms and reversible vasogenic edema, most often associated with hypertension, renal dysfunction, and exposure to immunosuppressive therapies. Endothelial injury and blood-brain barrier disruption are central to its pathogenesis [1-3].

Rituximab, an anti-CD20 monoclonal antibody widely used in ANCA-associated vasculitis, is generally well tolerated but has rarely been implicated in PRES. Most reported cases occur in the setting of active autoimmune disease, renal impairment, or concomitant immunosuppression, limiting causal attribution [3,4].

We describe a patient with hydralazine-induced dual-positive ANCA-associated vasculitis who developed PRES within hours of rituximab infusion in the absence of renal dysfunction or additional induction agents. This case highlights a rare but serious complication of rituximab and underscores the need for heightened neurologic and blood pressure monitoring in patients with active or drug-induced ANCA-associated vasculitis.

## Case presentation

A 59-year-old woman with a history of hypertension, type 2 diabetes mellitus, Graves’ disease status post radioactive iodine ablation (2015), right base-of-tongue squamous cell carcinoma treated with cisplatin-based chemoradiation (2022), and prior methimazole-induced ANCA-associated vasculitis with pulmonary involvement (2015) presented to the emergency department with one week of dyspnea and cough, which had progressed to hemoptysis over the preceding three days. She also reported swelling, pain, and morning stiffness lasting 1-2 hours in the fingers and wrists. She denied sicca symptoms, mucosal ulcers, visual changes, alopecia, Raynaud’s phenomenon, rash, gastrointestinal symptoms, or constitutional complaints. Family history was notable for systemic lupus erythematosus in the two sisters. 

Three months prior, hydralazine had been initiated for blood pressure control. Several weeks later, she developed fatigue and polyarthralgia. Antibody testing at that time revealed a positive antinuclear antibody (ANA), anti-double-stranded DNA (anti-dsDNA), rheumatoid factor (RF), SSA, and anti-histone antibodies. 

On arrival, she was afebrile (37.2 °C) with a heart rate of 107 bpm, blood pressure of 187/95 mmHg, respiratory rate of 26 breaths/min, and oxygen saturation 97% on room air. Examination revealed synovitis, decreased range of motion, and tenderness of the bilateral MCP, PIP, and wrist joints. Pulmonary auscultation demonstrated rales and rhonchi bilaterally. 

Investigations 

Laboratory evaluation showed leukopenia (2.5 × 10³/µL), normocytic anemia (hemoglobin 9.7 g/dL), elevated ESR (47 mm/hr) and CRP (17.2 mg/L), and normal renal and hepatic function. Urinalysis revealed 3+ blood, 6-10 red blood cells/hpf, and subnephrotic proteinuria (<1 g/day). The urine drug screen was negative for cocaine. She endorsed a history of painless hematuria with a negative renal biopsy in 2015. Chest CT demonstrated multifocal patchy bilateral solid opacities and mild bibasilar atelectasis (Tables 1, 2). Bilateral hand radiographs revealed multifocal erosions involving the intercarpal spaces and the base of the first metacarpals. Serologic evaluation was notable for positive myeloperoxidase (MPO) (>8), proteinase-3 (PR3) (2.1), anti-dsDNA, anti-histone, ANA, RF, and SSA, with low C3 and normal C4. Anti-cyclic citrullinated peptide (anti-CCP), anti-glomerular basement membrane (GBM), and antiphospholipid antibodies were negative, and CH50 was normal. Extensive infectious testing for pulmonary pathogens, including Histoplasma, Legionella, Mycoplasma, Streptococcus pneumoniae, Aspergillus, tuberculosis, and Blastomyces, was negative. Hemolysis studies were unremarkable. 

**Table 1:** Routine Laboratory and Urinalysis Values

Test (Unit)	Observed Value	Normal Range
White Blood Cell Count (×10³/µL)	2.5	4.0–11.0
Hemoglobin (g/dL)	9.7	12–16
Mean Corpuscular Volume (MCV, fL)	Normocytic	80–100
Erythrocyte Sedimentation Rate (ESR, mm/hr)	47	0–20
C-Reactive Protein (CRP, mg/L)	17.2	0–5
Blood Urea Nitrogen (BUN, mg/dL)	12	7–20
Creatinine (mg/dL)	0.8	0.6–1.3
Aspartate Aminotransferase (AST, U/L)	33	10–40
Alanine Aminotransferase (ALT, U/L)	48	7–56
Urinalysis – Blood	3+	Negative
Urinalysis – RBCs per High-Power Field (RBCs/hpf)	6–10	0–3
Urinalysis – Protein	<1 g/day	<150 mg/day

**Table 2:** Serologic and Antibody Laboratory Values RBCs: red blood cells; MPO: myeloperoxidase; PR3: proteinase-3; ANA: antinuclear antibody; RF: rheumatoid factor; SSA: Sjögren’s-syndrome-related antigen A; Anti-CCP: anti-cyclic citrullinated peptide; GBM: glomerular basement membrane; CH50: total complement activity

Antibody/Complement	Observed Value	Normal Range
Myeloperoxidase (MPO) Antibody	>8	Negative
Proteinase-3 (PR3) Antibody	2.1	Negative
Anti-dsDNA	Positive	Negative
Anti-Histone	Positive	Negative
Antinuclear Antibody (ANA)	Positive	Negative
Rheumatoid Factor (RF)	Positive	Negative
SSA Antibody	Positive	Negative
Anti-Cyclic Citrullinated Peptide (Anti-CCP)	Negative	Negative
Anti-Glomerular Basement Membrane (Anti-GBM)	Negative	Negative
Antiphospholipid Antibodies	Negative	Negative
C3 Complement (mg/dL)	Low	90–180
C4 Complement (mg/dL)	Normal	10–40
Total Complement Activity (CH50, U/mL)	Normal	31–60

In the emergency department, she developed hypoxia requiring 6 L/min oxygen via the nasal cannula. High-dose steroids were initiated (1 mg/kg/day), and hydralazine was discontinued. Given the recent hydralazine exposure, hemoptysis with imaging suggestive of diffuse alveolar hemorrhage, dual-positive MPO and PR3 ANCA, and her prior methimazole-induced ANCA vasculitis, hydralazine-induced dual-positive ANCA vasculitis was diagnosed. Concomitant leukopenia, hematuria, inflammatory arthritis, positive ANA and anti-dsDNA, elevated RF, and a Clinical Disease Activity Index (CDAI) of 19.3 raised concern for SLE-RA overlap syndrome. 

Over the next 24 hours, she demonstrated remarkable improvement: her hemoptysis resolved and her oxygen requirement decreased to 4 L/min. However, within the subsequent 48 hours, she developed acute worsening hypoxia necessitating endotracheal intubation. Pulse-dose glucocorticoids were initiated, and management proceeded per the RAVE protocol with planned weekly rituximab infusions for four doses and a total of seven plasmapheresis sessions over 14 days. On the first day of intubation, she received methylprednisolone 1 g IV twice daily for three days, followed by her first rituximab infusion and an immediate plasmapheresis session. The patient’s family refused a bronchoscopy. 

**Figure 1:**
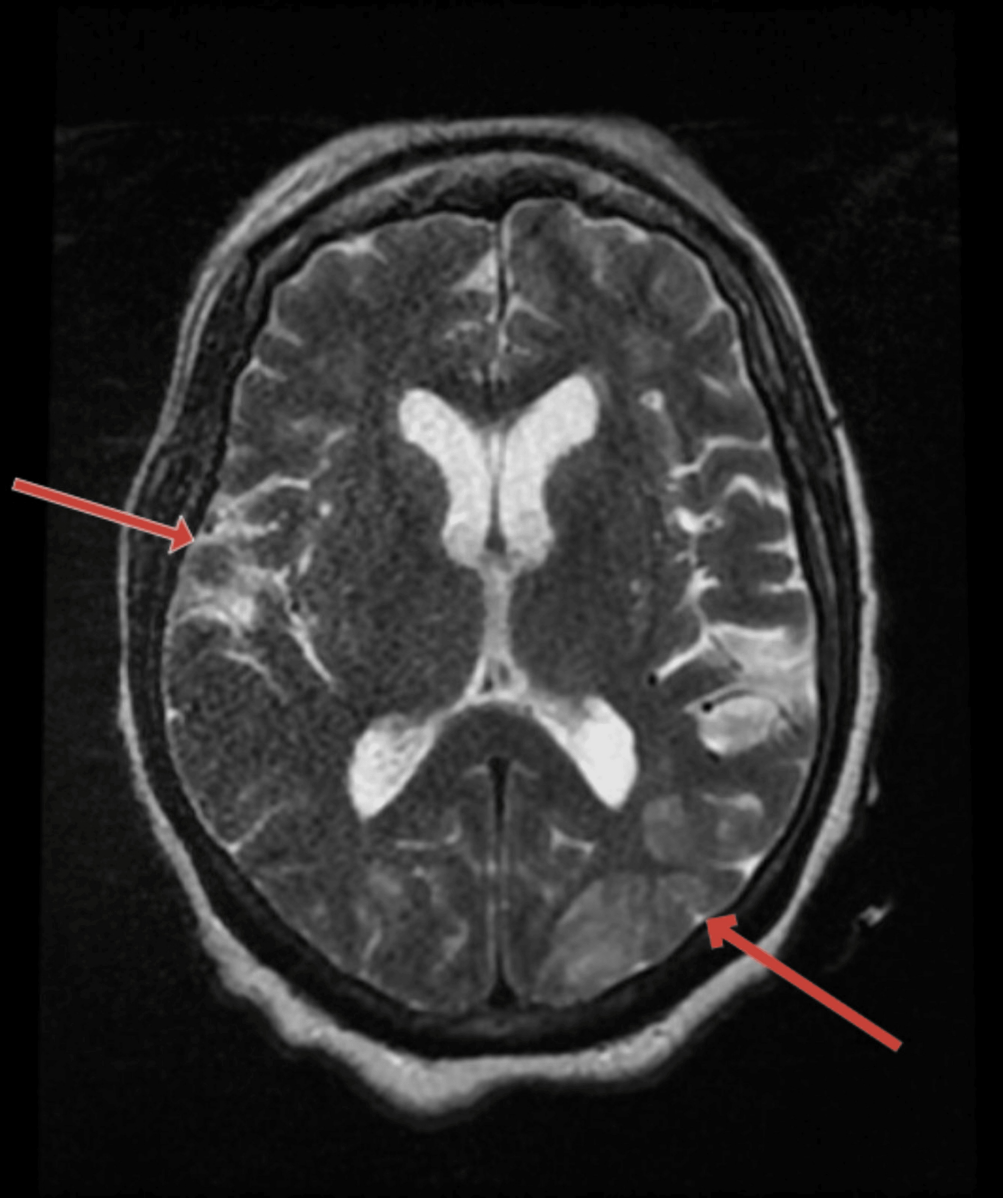
MRI Brain Showing Confluent T2 FLAIR Hyperintensity in the Bilateral Parietal and Occipital Lobes (Red Arrows) FLAIR: fluid attenuated inversion recovery

One week later, within 6-8 hours of her second rituximab infusion, she acutely developed severe hypertension (blood pressure 199/100 mmHg), new right-sided gaze deviation, horizontal nystagmus, and flaccid extremities. A non-contrast head CT revealed bilateral parietal and occipital hypodensities. Continuous EEG monitoring demonstrated a 50% seizure burden, which resolved following administration of levetiracetam and lorazepam. Blood pressure was controlled with intravenous labetalol and a nicardipine infusion. Brain MRI subsequently confirmed the diagnosis of PRES (Figure 1).

Rituximab was discontinued indefinitely. She was maintained on prednisone 40 mg daily, completed all seven planned plasmapheresis sessions, and was successfully extubated. The patient and her family declined further immunosuppressive therapy, including cyclophosphamide. She didn't have any long-term neurological deficits. 

## Discussion

PRES is a clinical and radiological entity first described by Hinchey et al. [1], characterized by reversible subcortical vasogenic edema predominantly affecting the white matter of the posterior cerebral lobes. Rituximab, an anti-cluster of differentiation 20 (anti-CD20) monoclonal antibody, is increasingly used in hematological malignancies and autoimmune conditions, including ANCA-associated vasculitis. While common adverse effects include infusion reactions, infections, and cytopenias, rare complications such as progressive multifocal leukoencephalopathy, cytokine release syndrome, respiratory failure, and PRES have been reported. We describe a patient with drug-induced ANCA vasculitis who developed PRES shortly after receiving rituximab.

PRES in patients with systemic lupus erythematosus or vasculitis treated with rituximab is exceedingly rare, with only isolated case reports and small series documented [2-4]. Risk factors include the underlying autoimmune disease, exposure to immunomodulatory agents (notably rituximab), concomitant immunosuppressants, renal dysfunction, severe or labile hypertension, and recent transplantation. The pathophysiology involves cerebrovascular endothelial dysfunction, with rituximab and other immune-modifying agents potentially triggering endothelial injury, breakdown of the blood-brain barrier, and vasogenic edema. Chronic immune activation in autoimmune disease, along with elevated circulating cytokines such as tumor necrosis factor-alpha (TNF-α), interleukin-1 (IL-1), and endothelin-1, further disrupts endothelial tight junctions and increases vascular permeability [5,6].

Although PRES has been described during active vasculitis and after cyclophosphamide or high-dose corticosteroids, its occurrence with rituximab is rare. The first well-documented case in ANCA-associated vasculitis was reported by Wardrope et al., in which PRES developed within hours of rituximab infusion [7]. Subsequent reports by Kim et al. [8], Dong et al. [9], and Ando et al. [10] describe PRES in patients treated with rituximab for microscopic polyangiitis or granulomatosis with polyangiitis; however, delayed onset (more than two weeks), comorbid hypertension, renal impairment, and disease activity often confounded causality. Collectively, the literature suggests that while rituximab-related PRES can occur, most cases are multifactorial, with active vasculitis, renal failure, and blood pressure fluctuations contributing.

In our patient, hypertension was well-controlled with antihypertensive medications prior to rituximab; she had no renal dysfunction and received no other immunosuppressants aside from rituximab and high-dose corticosteroids. Within hours of rituximab infusion, she developed uncontrolled hypertension, confusion, and seizures, which were successfully managed with intravenous lorazepam and nicardipine. The patient and her family chose to continue treatment with high-dose corticosteroids alone, without an additional induction agent. The temporal relationship strongly implicates rituximab as the trigger for PRES, making this a rare and noteworthy case. This case underscores that patients with drug-induced ANCA vasculitis are also at risk of PRES following rituximab, similar to patients with other autoimmune diseases or post-transplant immunosuppression. Clinicians should maintain a high index of suspicion, closely monitor blood pressure, and observe neurological status during and after rituximab infusion in this population. Early recognition and prompt management are essential to prevent morbidity and ensure reversibility of PRES.

## Conclusions

This case highlights an uncommon but important complication of rituximab therapy, PRES, in the setting of severe hydralazine-induced ANCA-associated vasculitis. It underscores the need for clinicians to maintain a high index of suspicion for PRES, particularly in patients with active vasculitis, severe systemic inflammation, or unstable blood pressure. The development of acute neurologic symptoms after rituximab infusion, such as visual disturbances, gaze deviation, seizures, or altered mental status, should trigger immediate discontinuation of therapy and prompt evaluation for PRES. Close monitoring before, during, and after rituximab administration is essential to ensure timely recognition and management of this potentially reversible but serious complication.
